# The *Jeff* Mouse Mutant Model for Chronic Otitis Media Manifests Gain-of-Function as Well as Loss-of-Function Effects

**DOI:** 10.3389/fgene.2020.00498

**Published:** 2020-05-19

**Authors:** Oana Kubinyecz, Pratik P. Vikhe, Thomas Purnell, Steve D. M. Brown, Hilda Tateossian

**Affiliations:** Mammalian Genetics Unit, MRC Harwell Institute, Harwell, United Kingdom

**Keywords:** otitis media, chronic otitis media, FBXO11, mouse model, mutation

## Abstract

Chronic otitis media (OM) is the most common cause of hearing loss worldwide, yet the underlying genetics and molecular pathology are poorly understood. The mouse mutant *Jeff* is a single gene mouse model for OM identified from a deafness screen as part of an ENU mutagenesis program at MRC Harwell. *Jeff* carries a missense mutation in the *Fbxo11* gene. *Jeff* heterozygotes (*Fbxo11^*Jf/+*^)* develop chronic OM at weaning and have reduced hearing. Homozygotes (*Fbxo11*^*Jf/Jf*^) display perinatal lethality due to developmental epithelial abnormalities. In order to investigate the role of FBXO11 and the type of mutation responsible for the phenotype of the *Jeff* mice, a knock-out mouse model was created and compared to *Jeff*. Surprisingly, the heterozygote knock-outs (*Fbxo11*^*tm2b/+*^) show a much milder phenotype: they do not display any auditory deficit and only some of them have thickened middle ear epithelial lining with no fluid in the ear. In addition, the knock-out homozygote embryos (*Fbxo11*^*tm2b/tm2b*^), as well as the compound heterozygotes (*Fbxo11*^*tm2b/Jf*^) show only mild abnormalities compared to *Jeff* homozygotes (*Fbxo11*^*Jf/Jf*^). Interestingly, 3 days after intranasal inoculation of the *Fbxo11*^*tm2b/+*^ mice with non-typeable *Haemophilus influenzae* (NTHi) a proportion of them have inflamed middle ear mucosa and fluid accumulation in the ear suggesting that the *Fbxo11* knock-out mice are predisposed to NTHi induced middle ear inflammation. In conclusion, the finding that the phenotype of the *Jeff* mutant is much more severe than the knock-out indicates that the mutation in *Jeff* manifests gain-of-function as well as loss-of-function effects at both embryonic and adult stages.

## Introduction

Otitis media (OM) is an inflammatory disease of the middle ear. Otitis media with effusion (OME) is a type of OM that is caused by a build-up of fluid within the middle ear and results in conductive hearing impairment. It is most common in young children. When the inflammation persists for longer it is considered as chronic otitis media with effusion (COME). COME is a multifactorial disease with a significant impact on language development and behavior. It is also an indication for a common surgical treatment (tympanostomy) at an early age in developed countries (Kubba et al., 2000). Until recently, not much was known about the underlying cellular mechanisms leading to OM. However, a deafness screen as part of a larger scale ENU mouse mutagenesis program (Nolan et al., 2000) identified three mouse models that display conductive deafness due to the development of COME: *Jeff* (Hardisty et al., 2003), *Junbo* (Parkinson et al., 2006), and *edison* mice (Crompton et al., 2017) and throw light on the genes and pathways involved in susceptibility to OM.

*Jeff* is a semi-dominant mutant that in heterozygotes displays conductive hearing loss caused by the development of chronic suppurative OM at weaning age (Hardisty et al., 2003). The *Jeff* mice are smaller than their wild-type littermates and have mild craniofacial abnormalities. The homozygotes exhibit perinatal lethality due to respiratory problems, cleft palate and eyelids open at birth (Hardisty-Hughes et al., 2006). The lungs of homozygote embryos are severely affected, with a smaller average airway width and significantly lower number of airways (Tateossian et al., 2009).

The gene mutated in *Jeff* mice is *Fbxo11*, a member of the F-box family (Hardisty-Hughes et al., 2006). It is located on chromosome 17 and has two protein-coding isoforms. The mutation consists of a single base transversion, from A to T in exon 13 of the *Fbxo11* gene, causing a glutamine to leucine change in a highly conserved region of the protein (Hardisty-Hughes et al., 2006). Another point mutation in exon 7 of the *Fbxo11* gene, named *Mutt*, was identified from the same screen. It leads to a serine to leucine change, within another conserved region of the protein. A proportion of *Mutt* heterozygotes showed mild craniofacial abnormality (57%) and reduced startle response (13%) with no OM at the age of 2 months, suggesting that *Mutt* is a weaker hypomorphic allele of *Fbxo11* in comparison to the *Jeff* mutation. In addition, a large proportion (83%) of the *Mutt* homozygotes survive in comparison to the 100% lethality found in *Jeff* homozygotes, which also underlines the hypomorphic nature of the *Mutt* allele. The surviving *Mutt* homozygote mice, demonstrate short face (84%) and reduced startle response (42%) in the absence of OM (Hardisty-Hughes et al., 2006).

In order to determine if the point mutation in *Jeff* mice is a loss-of-function or a gain-of-function mutation, a knock-out strain of *Fbxo11* was created. We studied the phenotype of the heterozygote (*Fbxo*11^*tm*2*b*⁣/ +^) and homozygote (*Fbxo*11^*tm*2*b*/*tm*2*b*^) knock-out mice and also the compound heterozygotes (*Fbxo*11^*tm*2*b*/*Jf*^) in order to compare them with the phenotype of the *Jeff* mice (both *Fbxo11*^*Jf/+*^ and *Fbxo11*^*Jf/Jf*^). Here we report that the phenotype of the *Jeff* mutant is much more severe than the phenotype of the *Fbxo11* knock-out mouse. The heterozygote knock-outs (*Fbxo*11^*tm*2*b*⁣/ +^) do not display OM but some of the homozygotes (*Fbxo*11^*tm*2*b*/*tm*2*b*^) as well as some of the compound mutants (*Fbxo*11^*tm*2*b*/*Jf*^) show cleft palate abnormalities, a much milder phenotype compared to *Jeff* mice. The result supports the conclusion that the mutation in *Jeff* manifests gain-of-function as well as loss of function effects at both embryonic and adult stages.

## Materials and Methods

### Mice Husbandry

*Fbxo11* knock-out mice [*Fbxo*11^*tm*2*b*(*EUCOMM*)*Wtsi*^] were produced by the European Conditional Mouse Mutagenesis Program at Harwell (Friedel et al., 2011; Skarnes et al., 2011; Bradley et al., 2012). The heterozygotes (*Fbxo*11^*tm*2*b*⁣/ +^) were generated on and maintained on a pure C57BL/6NTac background. The *Jeff* colony was maintained on a mixed C3H/HeH-C57BL/6J background because they do not survive on a congenic C57BL/6J background. The compound mutant embryos (*Fbxo*11^*tm*2*b*/*Jf*^) were generated on a mixed C57BL/6NTac and C3H/HeH-C57BL/6J background. All animal experimentation was approved by the Animal Welfare and Ethical Review Body at MRC, Harwell. The humane care and use of mice in this study was under the authority of the appropriate United Kingdom Home Office Project License.

### Genotyping

The *Jeff* mice were genotyped as previously described (Hardisty-Hughes et al., 2006).

For the genotyping of the *Fbxo11* knock-out mice a qPCR based genotyping strategy was used. The following primers and probes were used:

Primers for the wild-type allele Fbxo11-CR-LOA: forward, 5′-TTGCTGGAACAAGACCTTTGTAG-3′ and reverse, 5′-GGCAACAGGAGCTATCACTCA-3′. FAM labeled probe: 5′-AGCTGCTTGCGTGTGTAAACGC-3′.
Primers for the LacZ assay: LacZ forward, 5′- CTCGCCACTTCAACATCAAC-3′ and reverse, 5′- TTATCAGCCGGAAAACCTACC-3′. FAM labeled probe: 5′- TCGCCATTTGACCACTACCATCAATCC-3′.

DNA was extracted from ear clips using Applied Biosystems^TM^ TaqMan Sample-to-SNP Kit (4403313, Applied Biosystems^TM^). Reaction mixtures (10 μL) contained 5 μL TaqMan GTXpress^TM^ master mix (4401892, Applied Biosystems^TM^), 0.225 μL 20 μM from each primer, 0.3 μL 15 μM probe, 2.5 μL 10 times diluted DNA extract and water. The samples were amplified (95°C for 20 s, followed by 40 cycles of 95°C for 3 s and 60°C for 3 s) and the results were analyzed using CopyCaller Software v2.0 from Applied Biosystems.

### Histology

Adult and embryonic heads, lungs or whole bodies (embryonic stages E15.5; E18.5), were collected and fixed in 10% buffered formaldehyde, decalcified and embedded in paraffin following routine procedures. 5 μM-thick sections were obtained and stained with hematoxylin and eosin for morphological observations. Goblet cells were identified by a combined Alcian blue/Periodic acid-Schiff staining method (AB-PAS) staining method.

### Auditory Brainstem Response (ABR)

One and two-months–old mice were anesthetized (ketamine hydrochloride, 100 mg/kg; xylasine, 10 mg/kg) and placed on a heated mat in a sound attenuating chamber. Acoustic stimuli were delivered to the right ear, from a distance of 1.5 cm, via a free field transducer controlled by TDT SinGen/BioSig software. ABR responses were collected, amplified and averaged using the BioSig software. Broadband click stimuli were presented at 90 dB SPL and gradually decreased in steps of 5 dB until a threshold was visually determined by the lack of replicable response peaks. The test was analyzed as previously described (Hardisty-Hughes et al., 2010).

### Tissue Collection and Preparation for Western Blot

Embryos were collected and transferred in cold PBS containing protease inhibitor cocktail (04 693 124 001, Roche). Lungs were dissected out, homogenized in extraction buffer (1% NP_–_40, 1M Tris, 1M NaCl; pH 8, protease and phosphatase inhibitor cocktails) in Precellys homogenizers for 20 s, and after centrifugation (10 min, 10,000 rpm, 4^°^C) the protein concentration of the supernatant was determined using DC^TM^ Protein Assay kit (500–0116, Bio-Rad).

### Western Blot Analysis

Lysates were resolved in NuPAGE^TM^ 7% Tris-Acetate Gel (EA03555, Invitrogen), blotted onto a nitrocellulose membrane and incubated with two anti-FBXO11 antibodies: A301-177A and A301-178A, Bethyl Laboratories) in 1:1000 dilutions. ECL Plus system was used (32132, Thermo Scientific, Pierce^TM^) for blot detection using X-ray film. Anti-rabbit IgG-HRP conjugated antibody was used as a secondary antibody (170-6515, Bio-Rad) and actin (A2066, Sigma) was used as a loading control.

### Immunohistochemistry

Paraffin sections were de-waxed in xylene substitute and rehydrated via graded ethanol solutions. Endogenous peroxidase was blocked with 3% hydrogen peroxide in isopropanol for 30 min. Heat-induced epitope retrieval was performed using a microwave. Sections were incubated overnight with primary antibodies against: Cleaved Caspase-3 (Asp175) (5A1E); 1:1000 (9664, Cell Signaling Technology); F4/80, 1:200 (MF48005, Invitrogen); myeloperoxidase, 1:200 (ab9535, Abcam) and NTHi162sr, 1:10000, custom made (Vikhe et al., 2019). The Vectastain Elite ABC HRP kit (PK-6101, Vector Laboratories) kit was used according to the manufacturer’s instructions for all of the antibodies except for F4/80 for which a goat anti rat HRP, 1:200, was used (7077S, Cell Signaling Technology). For development of the signal, the DAB + chromogen system was used (K3468, DAKO). Counterstaining was carried out with hematoxylin.

### Intranasal Inoculation

Two-months-old wild-type littermates, C57BL/6NTac (*n* = 11), and heterozygote knock-out mice (*n* = 12), male and female, were inoculated as described previously (Hood et al., 2016). Briefly, mice were inoculated intranasally under gas anesthesia with 5 μL per nares of NTHi 162sr (streptomycin resistant) cell suspension at a concentration of 10^8^ CFU/mL in PBS–1% gelatin. After 3 days of the challenge the mice were euthanized, half of the animals were used to collect the ears for histological analyses and from the other half, the middle ear fluid was harvested for culture. For the culturing the fluid was collected into PBS buffer (for wild-type mice the ears were just washed with PBS buffer), plated on streptomycin BHI (Brain Heart Infusion) plates with streptomycin and the bacterial colony count was obtained after overnight incubation at 37;C.

### Data Analysis

We used the Chi-squared test to compare the difference between the observed and the expected number of the mutant mice from crosses. Two-tailed *t*-test was used for comparing mean ABR thresholds and mucoperiosteal thickness. A value of *p* < 0.05 was considered significant.

## Results

### Generation of the *Fbxo11* Knock-Out Mice

**FIGURE 1:**
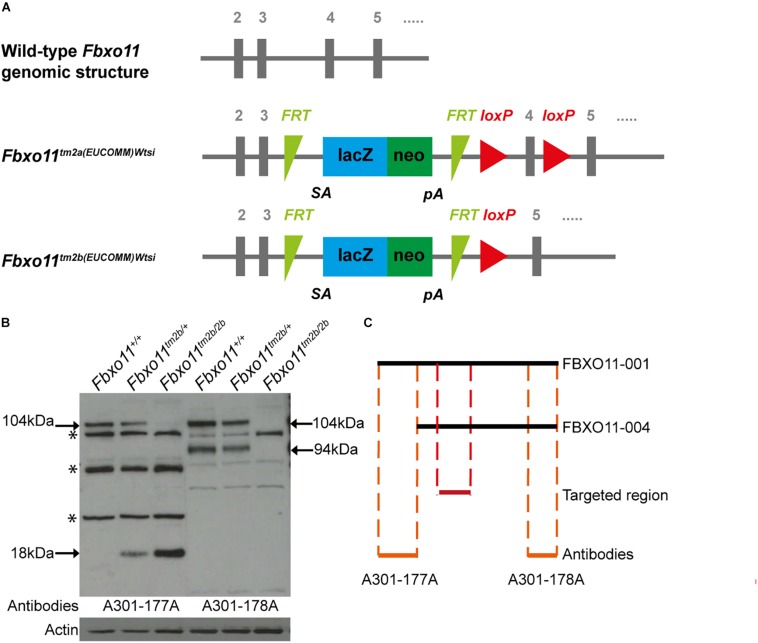
Generation of the *Fbxo11* knock-out mice. **(A)** Schematic representation of the knock-out strategy used by EUCOMM to produce the mice (not to scale), illustrating the wild type structure of the *Fbxo11* gene from exons 2–5 and the structure of the mutant tm2a allele generated (see results). The tm2b allele is derived by cre excision of the tm2a allele, deleting exon 4. **(B)** Protein levels of FBXO11 in E18.5 embryonic lungs. Neither the 177A antibody which detects the large isoform of mouse FBXO11 (FBXO11-001, 930 aa, 104 kDa) nor the 178A, which detects both the large and small isoforms (FBXO11-001, 930 aa, 104 kDa and FBXO11-004, 855 aa, 94 kDa) detected the protein in the homozygote embryonic lung tissue. Actin was used as loading control. Truncated protein, 18 kDa, was detected in the mutant tissues. Cross- reactive bands are indicated by an asterisk. **(C)** Alignment of the two FBXO11 isoforms (FBXO11-001 and FBXO11-004), the fragments of FBXO11 protein used to produce the A301-177A and A301-178A antibodies for FBXO11 and the targeted region in the knock-out (not to scale).

The promoterless EUCOMM (European Conditional Mouse Mutagenesis Program) tm2a vector (PGS00030_B_D03) was used to generate a targeted knock-out first JM8A1.N3 embryonic stem (ES) cell clones (Friedel et al., 2011; Skarnes et al., 2011; Bradley et al., 2012). ES cell to mouse conversion (ES cell clone EPD248_1_H03) was carried out at MRC Harwell to generate chimeras, from which germline transmission of the *Fbxo*11^1*tm*2*a*(*EUCOMM*)*Wtsi*^ allele was achieved. The floxed critical region containing *Fbxo11* exon 4 (ENSMUSE00000539085) is excised by cre recombinase to yield the lacZ-tagged null *Fbxo*11^*tm*2*b*(*EUCOMM*)*Wtsi*^ allele (Figure 1A).

To confirm that the gene was successfully knocked-out we used western blots to measure the protein levels in mouse embryonic lungs. The FBXO11 mouse protein has two main protein-coding isoforms, 104 and 94 kDa. We used two antibodies; A301-178A which can recognize both isoforms and A301-177A which is specific for the full length “canonical” sequence. We detected reduced levels of FBXO11 in the heterozygote lung and no protein present in the homozygote embryonic lung (Figures 1B,C and Supplementary Figure S1). We also detected a small band (18 kDa) in the mutant tissues corresponding to the truncated protein, which was only present in the heterozygote and homozygote. In addition a band was detected between the two main FBXO11 isoforms with both antibodies in all three samples, wild-type, heterozygote and homozygote. This cross-reactive product was previously observed by us using the same antibodies (Tateossian et al., 2015) and has been noted by others (Abbas et al., 2013).

### Phenotype of the *Fbxo11*^*tm2b/+*^ Mice

**FIGURE 2:**
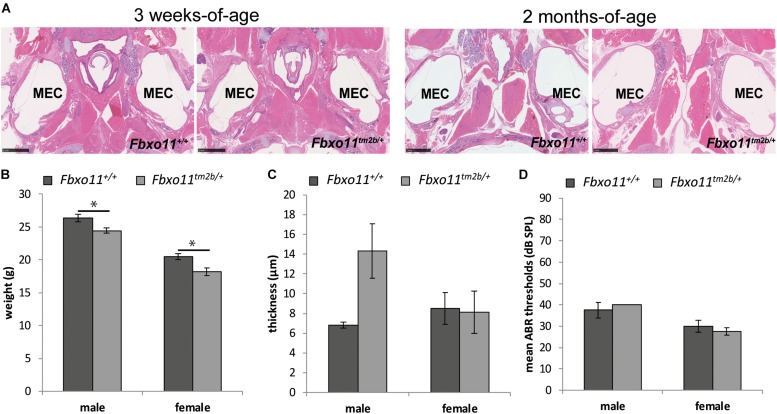
Phenotype of the *Fbxo*11^*tm*2*b*⁣/ +^ mice. **(A)** Hematoxylin-eosin stained transverse sections through the middle ear of 3-weeks and 2-months-old mice showing air-filled middle ear cavity for both heterozygote *Fbxo*11^*tm*2*b*⁣/ +^ and wild-type *Fbxo11*^+/+^ mice. Scale bars: 1 mM. MEC: middle ear cavity. **(B)** Comparison of the weight of the mice at the age of 2-months. The weights were taken from six mice from each sex and genotype except for the female heterozygote mice for which we had seven mice. **(C)** Comparison of the thickness of the epithelial lining of the middle ear of 2-months-old mice. The measurements were taken from three mice (six ears) from each genotype. **(D)** Broadband click stimuli ABR thresholds in the right ears of 2-months-old wild-type *Fbxo11*^+/+^ and heterozygote *Fbxo*11^*tm*2*b*⁣/ +^ mice. Two wild-type males, three wild-type females, two heterozygote males and three heterozygote females were used for the test. As the data was exactly the same (40dB) for both heterozygote males used for this study, the error bars are not well visible in the graph. Bars: standard error of mean. *P*-values were determined using two-tailed *t*-test, ^∗^*p* ≤ 0.05.

To investigate the phenotype of the heterozygote knock-out mice (*Fbxo*11^*tm*2*b*⁣/ +^) we out-crossed them to C57BL/6NTac wild-type mice. We had only 37% *Fbxo*11^*tm*2*b*⁣/ +^ mice at weaning age (68/184) which was less than the expected 50% (*p* = 0.0004). We found that some pups were lost shortly after birth and prior to weaning. The surviving mice demonstrated a milder phenotype compared to *Jeff* mice (*Fbxo11*^*Jf/+*^). Similar to *Jeff* mice, both males and females were significantly smaller than their wild-type littermates at the age of 2 months (*p* = 0.022 for males, *p* = 0.011 for females; Figure 2B). However, unlike *Jeff* mice (*Fbxo11*^*Jf/+*^) they do not spontaneously develop OM. The Broadband click stimuli ABR test revealed that the *Fbxo*11^*tm*2*b*⁣/ +^ mice do not have significantly reduced hearing. The ABR thresholds of the *Fbxo*11^*tm*2*b*⁣/ +^ mice were comparable with the wild-type thresholds (Figure 2D). In addition the histological analysis of the middle ear of 3-weeks, 2- and 5-months-old *Fbxo*11^*tm*2*b*⁣/ +^ mutants showed no fluid in the ears (Figure 2A). We also measured the mucoperiosteal thickness of the middle ears of 3 weeks and 2-months-old *Fbxo*11^*tm*2*b*⁣/ +^ mice. We did not find any difference in the thickness of the middle ear epithelial lining between the two genotypes at the age of 3 weeks (*p* = 0.930 for males, *p* = 0.463 for females). The result was the same between 2-months-old female *Fbxo*11^*tm*2*b*⁣/ +^ mice and wild-types (*p* = 0.909). There was some difference in the mucosa thickness in male 2 months old *Fbxo*11^*tm*2*b*⁣/ +^ mice compared to wild-type littermates, however it was not significant (*p* = 0.0554; Figure 2C). Neither female, nor male *Fbxo*11^*tm*2*b*⁣/ +^ mice, displayed either a significant thickened middle ear epithelial lining or a reduced hearing phenotype. Thus, overall the phenotype of the *Fbxo*11^*tm*2*b*⁣/ +^ mice resembles more closely the phenotype of *Mutt* (*Fbxo11*^*Mutt/+*^) than *Jeff* mice (*Fbxo11*^*Jf/+*^).

### OM Phenotype of the *Fbxo*11^*tm*2*b*⁣/ +^ Mice After Inoculation With NTHi

**FIGURE 3:**
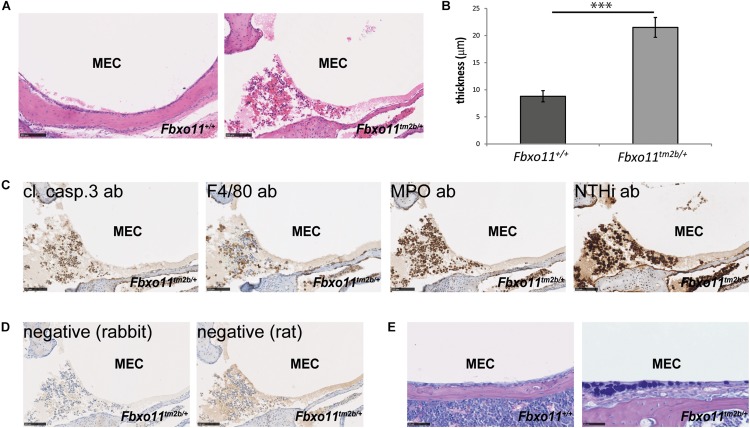
OM phenotype of the *Fbxo*11^*tm*2*b*⁣/ +^ mice after inoculation with NTHi. **(A)** Hematoxylin-eosin stained transverse sections through the middle ear of 2-months-old wild-type *Fbxo11*^+/+^ and heterozygote *Fbxo*11^*tm*2*b*⁣/ +^ mice after the challenge. Scale bars: 100 μM. **(B)** Comparison of the thickness of the epithelial lining of the middle ear for each genotype after the challenge. The measurements were taken from two mice (one male and one female, three ears) from each genotype. Bars: standard error of mean. *P*-values were determined using two-tailed *t*-test. ^∗∗∗^*p* ≤ 0.001. **(C)** Immunohistochemistry to detect different cell types in the middle ear fluid: cleaved caspase 3 antibody (cl. casp. 3) detecting apoptotic cells; F4/80 detecting foamy macrophages; myeloperoxidase antibody (MPO) detecting neutrophils and antibody against NTHi162sr (NTHi) detecting NTHi bacteria in the middle ear fluid. Scale bars: 100 μM. **(D)** Negative controls for the staining for antibodies raised in rat (for F4/80) or rabbit (all the other). Scale bars: 100 μM. **(E)** The AB-PAS staining detected goblet cells in the epithelial lining of the *Fbxo*11^*tm*2*b*⁣/ +^ mice middle ear cavity. Scale bars: 50 μM.

Two-month-old *Jeff* mice have been previously inoculated with NTHi162kr and it was discovered that 7 days post-inoculation they have middle ear titers of 2 × 10^2^ colony-forming units (CFU)/μL and infection rates of 15% (Hood et al., 2016). In this study we inoculated 2-months-old *Fbxo*11^*tm*2*b*⁣/ +^ mice and wild-type littermates with NTHi162sr, the middle ear fluid was collected at 3 days post-challenge, cultured and the NTHi titers were calculated. A shorter 3 days inoculation challenge was chosen because *Fbxo*11^*tm*2*b*⁣/ +^ mice do not have middle ear fluid and our earlier infection studies (Hood et al., 2016; Vikhe et al., 2019) have shown that NTHi does not infect wild-type middle ears without any fluid after intranasal challenge. Samples from two out of six *Fbxo*11^*tm*2*b*⁣/ +^ mice, 33% (two out of 12 ears, middle ear infection rate 16.7%) were positive for the bacteria with average 3.6 × 10^2^ CFU/μL. There was no bacterial growth on the plates from wild-type ears. Half of the inoculated mice were used for histological examination and immunohistochemistry with different antibodies. Hematoxylin- and eosin-stained sections of middle ear bulla showed thickened epithelial lining and fluid in two out of six mice (one male with unilateral OM and one female mouse with bilateral OM), 33%. The average mucoperiosteal thickness of the middle ears was 21.5 μM compared to 8.8 μM in wild-type mice (*p* = 1.061 E-06; Figures 3A,B). In addition we detected apoptotic cells using a cleaved caspase 3 antibody; foamy macrophages with an F4/80 antibody and neutrophils using myeloperoxidase as a marker. To localize NTHi bacteria in the middle ear we used an antibody against NTHi162sr. We detected bacteria in the epithelial lining and in the fluid of the challenged mice (Figures 3C,D). The AB-PAS staining detected a high density of goblet cells (mucus-producing cells) in the epithelial lining of the *Fbxo11*Fbxo*11^*tm*2*b*⁣/ +^* mice middle ear cavity (Figure 3E).

Similar to *Fbxo11*^*Jf/+*^, *Fbxo*11^*tm*2*b*⁣/ +^ mice had low but significant NTHi titers. However, *Jeff* mutants have already inflamed middle ears pre-inoculation whereas in *Fbxo*11^*tm*2*b*⁣/ +^ NTHi induces inflammation and fluid accumulation in the middle ear. This finding suggests that the *Fbxo11* knock-out mice are predisposed to NTHi induced middle ear inflammation.

### Phenotype of the Homozygote *Fbxo11*^*tm2b/tm2b*^

**FIGURE 4:**
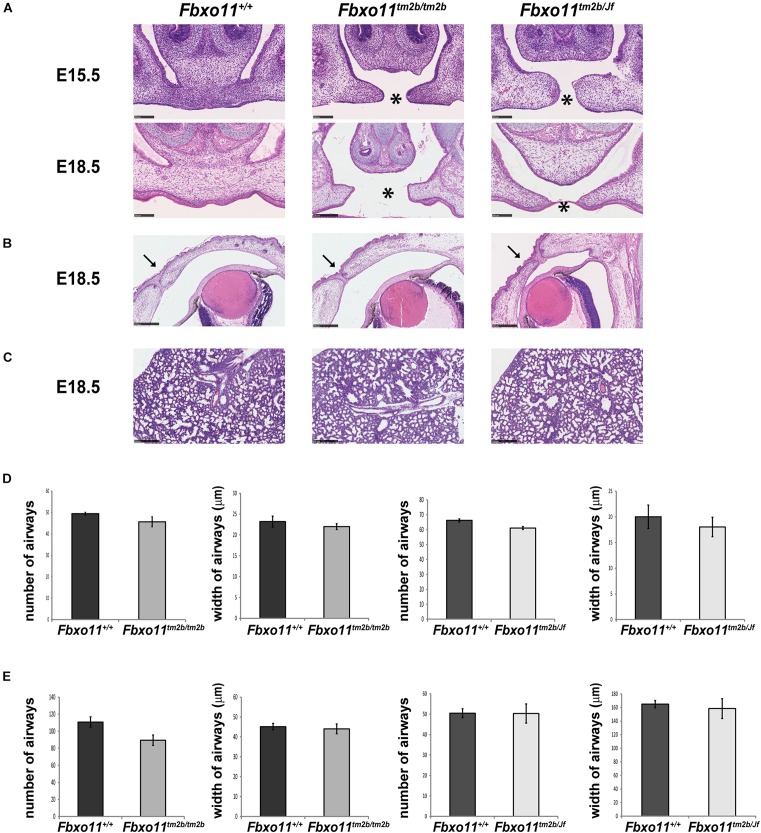
Phenotype of homozygote knock-out *Fbxo*11^*tm*2*b*/*tm*2*b*^ and compound heterozygote *Fbxo*11^*tm*2*b*/*Jf*^ embryos. **(A)** Hematoxylin-eosin stained coronal sections through the palate of E15.5 and E18.5 embryonic heads. Scale bars: 100 μM. The cleft palate is indicated by asterisk. **(B)** Hematoxylin-eosin stained coronal sections through the eyelids of E18.5 embryonic heads. Scale bars: 250 μM. Arrows indicate the fused eyelids. **(C)** Hematoxylin-eosin stained sections through the lungs of E18.5 embryos. Scale bars: 250 μM. **(D)** Comparison of the number of airways for three regions taken at random for three E15.5 individuals from each genotype and comparison of the width of 30 airways from three sections of embryonic lungs for three E15.5 individuals from each genotype. **(E)** Comparison of the number of airways for three regions taken at random for three E18.5 individuals from each genotype and comparison of the width of 30 airways from three sections of embryonic lungs for three E15.5 individuals from each genotype. Bars: standard error of mean.

Due to the fact that the *Jeff* homozygote mice (*Fbxo11*^*Jf/Jf*^) show perinatal lethality, only the embryonic phenotype of the *Fbxo11*^*tm2b/tm2b*^ mice was investigated. We collected embryos at stage E15.5 and E18.5. The homozygotes composed 20% of the embryos from heterozygote intercrosses at each stage, not significantly different from the expected 25 percent (5/25, *p* = 0.564 at E15.5 and 10/50, *p* = 0.414 at E18.5). At the developmental stage E15.5, when the palatal shelves are supposed to be already fused, 80% of the *Fbxo11*^*tm2b/tm2b*^ embryos (4/5) displayed a cleft, a phenotype similar with *Fbxo11*^*Jf/Jf*^ embryos. At stage E18.5 however, only 10% (1/10) had a cleft, and some presented abnormalities in the fusion (Figure 4A) indicating a delay in the palate fusion in the knock-out mice compared to wild-type mice. None of the E18.5 embryos had an eyelid open phenotype (Figure 4B). We previously reported that *Jeff* new born homozygote mice (*Fbxo11^*Jf/Jf*^)* have underdeveloped lungs (Tateossian et al., 2009) and thus investigated lung pathology in *Fbxo11*^*tm2b/tm2b*^ mice. However, unlike *Jeff* there was no significant difference in the number (*p* = 0.193) or the width (*p* = 0.431) of airways of the lungs of the *Fbxo11*^*tm2b/tm2b*^ embryos, compared to their wild-type littermates at either E15.5 or E18.5 (Figures 4C–E).

Similar to our findings with the knock-out heterozygote mice (*Fbxo11^*tm2b/+*^)*, the phenotype of the knock-out homozygote mice (*Fbxo11*^*tm2b/tm2b*^) was found to be much milder compared to *Fbxo11*^*Jf/Jf*^ mice.

### Phenotype of the Compound Heterozygote *Fbxo*11^*tm*2*b*/*Jf*^

We crossed mice heterozygous for *Jeff* (*Fbxo11*^*Jf/+*^) to *Fbxo*11^*tm*2*b*⁣/ +^ heterozygotes to produce compound mutants (*Fbxo*11^*tm*2*b*/*Jf*^). Corresponding with the expected ratio, 23.5% of the total embryos at E15.5 were genotyped as compound heterozygotes (*Fbxo*11^*tm*2*b*/*Jf*^), not significantly different from the expected 25% (4/17, *p* = 0.889). The percentage of the *Fbxo*11^*tm*2*b*/*Jf*^ compound mutants at E18.5 was less, 11.5%, but it was also not significantly less than the expected numbers (*p* = 0.075). At E15.5, 50% of the embryos presented cleft palate, and at E18.5 25% had cleft compared to the wild-type littermates (Figure 4A). The result suggested that in compound mutants there is a delay in the development of the palatal shelves. We did not detect any eyelid open phenotype or underdeveloped lungs in the compound mutants (Figures 4B–E).

## Discussion

*Jeff* mice are one of the first mouse models of OM (Hardisty et al., 2003). They carry a missense mutation in the *Fbxo11* gene (Hardisty-Hughes et al., 2006). In order to investigate the nature of the mutation in these mice we studied the phenotype of *Fbxo11* knock-out mice. We found that in comparison to *Jeff*, the *Fbxo11* knock-out mice appear less affected, with almost no significant differences between the wild-types and the mutants.

We have previously reported that *Jeff* heterozygotes develop OM at weaning age and the deafness phenotype is fully penetrant. They have inflamed middle ear mucosa, fluid in the middle ear and reduced hearing (Hardisty et al., 2003). We have also previously shown that the homozygotes have developmental abnormalities, cleft palate, eyelid open phenotype and perinatal lethality as a result of underdeveloped lungs (Hardisty-Hughes et al., 2006; Tateossian et al., 2009).

Surprisingly, *Fbxo11* knock-out heterozygotes (*Fbxo11*^*tm*2*b*⁣/ +^) demonstrate a much milder phenotype. They do not develop OM at any time point, and do not display any auditory deficit. However, we found that only 37% of the pups from heterozygote matings to wild type are heterozygotes (not 50%). Some of the pups were absent from the litters shortly after birth and they might account for the missing heterozygotes, thus suggesting that the knock-out influences the survival, through a yet unknown reason. The weight of the surviving mutants was recorded to be reduced at the age of 2-months which may contribute to the reduced survival rates of the mutants.

On a BL/6 background the otitis media phenotype and viability of the *Jeff* heterozygote mice (*Fbxo11*^*Jf/+*^) and the embryonic phenotype of the *Jeff* homozygote mice (*Fbxo11*^*Jf/Jf*^) are very severe and it was impossible to maintain the colony on this background. For this reason, the *Jeff* colony is maintained on a mixed C3H/HeH-C57BL/6J background. The heterozygote knock-out mice (*Fbxo*11^*tm*2*b*⁣/ +^) were generated on and maintained on an isogenic C57BL/6NTac background. Thus we analyzed the knock-out line on a severe BL/6 background. However, we cannot rule out potential differences in OM severity between a BL/6N vs. BL/6J background. What is notable is that on the BL/6N background the knock-out does not show any phenotypic indicators of OM.

*Jeff* mice (*Fbxo11*^*Jf/+*^) have been previously inoculated with NTHi and it was discovered that 7 days post-inoculation they have middle ear titers of 2 × 10^2^ CFU/μL and infection rates of 15% (Hood et al., 2016). To test if the knock-out heterozygote mice (*Fbxo*11^*tm*2*b*⁣/ +^) are susceptible to middle ear inflammation we performed intranasal inoculation with bacterial pathogen NTHi162sr. Three days post-challenge a third of the mice had inflamed middle ear lining and fluid in the ears, middle ear infection rate of 16.7%. Similar to *Jeff* mice (*Fbxo11*^*Jf/+*^) they also had low NTHi titers, 3.6 × 10^2^ CFU/μL. The fact that some of the *Fbxo*11^*tm*2*b*⁣/ +^ mice develop middle ear infection after the inoculation, irrespective of the absence of OM before the inoculation was very interesting. This finding indicates that the mutations in *Fbxo11* in both, *Jeff* and knock-out mice makes them predisposed to NTHi induced middle ear inflammation.

The embryonic development of the knock-out homozygotes (*Fbxo*11^*tm*2*b*/*tm*2*b*^) and compound mutants (*Fbxo*11^*tm*2*b*/*Jf*^) seems to be less affected than in the *Jeff* homozygotes (*Fbxo11*^*Jf/Jf*^). The only similarity is in the palatal shelf development. Mouse palatogenesis takes place between E11.5 and E15.5 (Ferguson, 1988). It is a process involving palatal shelf growth and elevation above the tongue followed by fusion of the shelves at about 15.5 embryonic days. The fact that at the embryonic stage E15.5, 80% of the null embryos (*Fbxo*11^*tm*2*b*/*tm*2*b*^) and 50% of the compound mutants (*Fbxo*11^*tm*2*b*/*Jf*^) have cleft, but at E18.5 most of the palatal shelves seem to be fused, indicates that there is a delay in development at E15.5 in both mutants, but this is corrected before E18.5. In addition, all the other developmental defects seen in *Jeff* are absent in *Fbxo*11^*tm*2*b*/*tm*2*b*^ and *Fbxo*11^*tm*2*b*/*Jf*^ embryos.

The mutation in *Mutt*, a weaker hypomorphic allele of *Fbxo11*, results mainly in a mild craniofacial defect in the mice (Hardisty-Hughes et al., 2006). Fifty-seven percent of *Mutt* heterozygotes showed mild craniofacial abnormality, a shortened face. A small proportion of *Mutt* homozygotes (17%) showed perinatal lethality, mild clefting of the palate and facial clefting. The phenotype of the *Fbxo11* knock-out mice looks very similar to the phenotype of *Mutt*.

In summary, the loss-of-function effects found in the *Fbxo*11^*tm*2*b*⁣/ +^ and *Fbxo*11^*tm*2*b*/*tm*2*b*^ mice appear very mild compared to *Jeff* heterozygotes (*Fbxo11*^+/Jf^) and homozygotes (*Fbxo11*^*Jf/Jf*^) respectively. This is also the case for the *Fbxo*11^*tm*2*b*/*Jf*^ compound heterozygote, which show a similar embryonic phenotype to *Fbxo*11^*tm*2*b*/*tm*2*b*^ mice. We conclude from this data that the *Jeff* mutant shows gain-of-function as well as loss-of-function effects, with the gain-of-function manifesting as the severe chronic otitis media displayed in the heterozygote and the cleft palate, eyelids open and lung phenotypes along with embryonic lethality displayed in the homozygotes. We were only able to investigate the *Fbxo*11^*tm*2*b*/*tm*2*b*^ and *Fbxo*11^*tm*2*b*/*Jf*^ embryonically and were not able to study the phenotype of adult mice. But we surmise that these mice would be potentially viable and that the *Fbxo*11^*tm*2*b*/*Jf*^ mice would demonstrate chronic otitis media. While these studies are focused on the *Fbxo11* knock-out and *Jeff* mutations and the nature of their pleiotropic effects across a range of tissues, including the middle ear, in order to better understand the pathways and mechanisms predisposing to middle ear inflammatory disease it will be important to develop and analyse the impact of these mutations exclusively in the middle ear using middle ear epithelial conditional mutants.

FBXO11 has a number of interacting partners and impacts on a number of pathways (Abida et al., 2007; Tateossian et al., 2009; Duan et al., 2012; Abbas et al., 2013; Rossi et al., 2013; Jin et al., 2015; Tateossian et al., 2015). FBXO11 is an E3 ubiquitin ligase, a substrate binding component of a SKP1-Cul1-Fbox protein complex involved in the post-translational modification of different target proteins. p53 has been shown to be neddylated by FBXO11 *in vitro* (Abida et al., 2007) and in the mouse developing lung (Tateossian et al., 2015). FBXO11 was reported to target BCL6 for ubiquitination and proteasomal degradation (Duan et al., 2012). Two studies demonstrated a role of FBXO11 in the ubiquitination and degradation of CDT2 (Abbas et al., 2013; Rossi et al., 2013). In addition SNAIL1/2 proteins were found to be recognized and ubiquitinated by FBXO11 (Jin et al., 2015). In our previous work we concluded that in the developing mouse FBXO11 regulates the TGFβ pathway (Tateossian et al., 2009) which is known to be critically involved with middle ear inflammation (Tateossian et al., 2013). This cross-talk may occur by interaction of FBXO11 with p53 (Tateossian et al., 2015). It is not inconceivable that mutations in FBXO11 might lead to gain-of-function effects, for example, leading to new interactions or strengthening existing interactions that would lead to dominant effects of the kind that we see in the *Jeff* mutant but not in a loss-of-function mutant. Both *in vivo* and *in vitro* characterization of the nature of interactions involving the mutant protein in *Jeff* mice may help to unravel precisely the nature of the gain-of-function effects that lead to the many changes we have already documented in relevant pathways that lead to impacts on TGFβ and p53 signaling in the *Jeff* mouse (Tateossian et al., 2009; Tateossian et al., 2015). Moreover, further work will be required to elucidate mechanisms associated with loss of function effects.

In conclusion, by comparing the phenotype of mice carrying a null mutation in the *Fbxo11* gene with the well-characterized chronic otitis media model, *Jeff*, we have demonstrated that the *Jeff* mutation is both a loss-of-function mutation and a gain-of-function mutation. This has important lessons for our further study of the molecular mechanisms by which FBXO11 elicits COME in both mice and the human population.

## Data Availability Statement

All datasets generated for this study are included in article/Supplementary Material.

## Ethics Statement

All animal experimentation was approved by the Animal Welfare and Ethical Review Body at MRC, Harwell.

## Author Contributions

OK carried out the phenotyping tests and analysis, contributed to the design of the study and the interpretation of the results, and participated in drafting the manuscript. PV and TP performed the inoculations and contributed to the interpretation of the results from these studies. HT and SB contributed to the design of the study, interpretation of results, and participated in drafting the manuscript. All authors read and approved the final manuscript.

## Conflict of Interest

The authors declare that the research was conducted in the absence of any commercial or financial relationships that could be construed as a potential conflict of interest.

## Supplementary Material

FIGURE S1 Original image files for the western blots included in Figure 1.
