# Supplementary figures and images for: The Jeff Mouse Mutant Model for Chronic Otitis Media Manifests Gain-of-Function as Well as Loss-of-Function Effects
Source: Front Genet. 2020 May 19;11:498. doi: 10.3389/fgene.2020.00498 (PMC7248398; doi:10.3389/fgene.2020.00498)

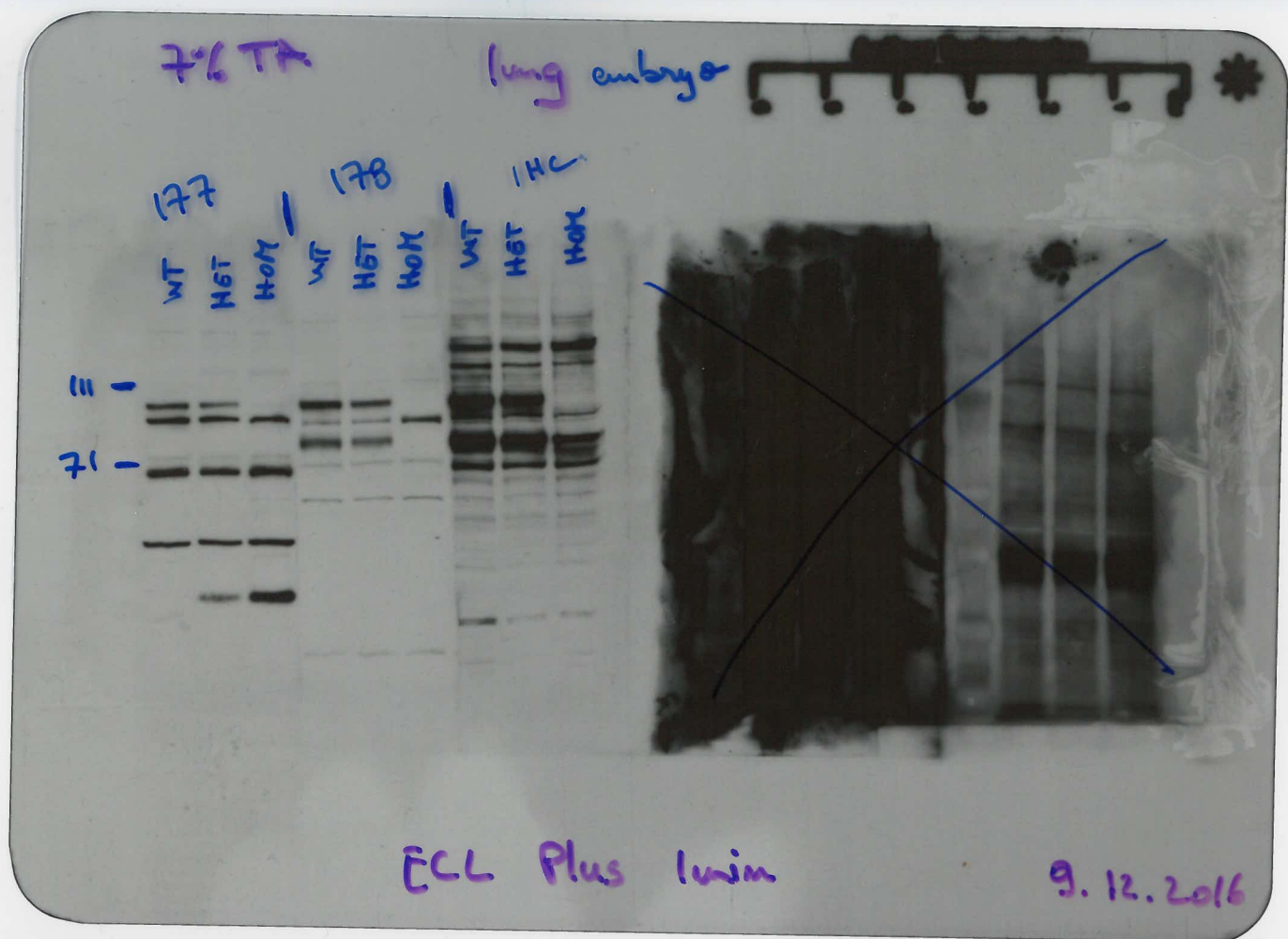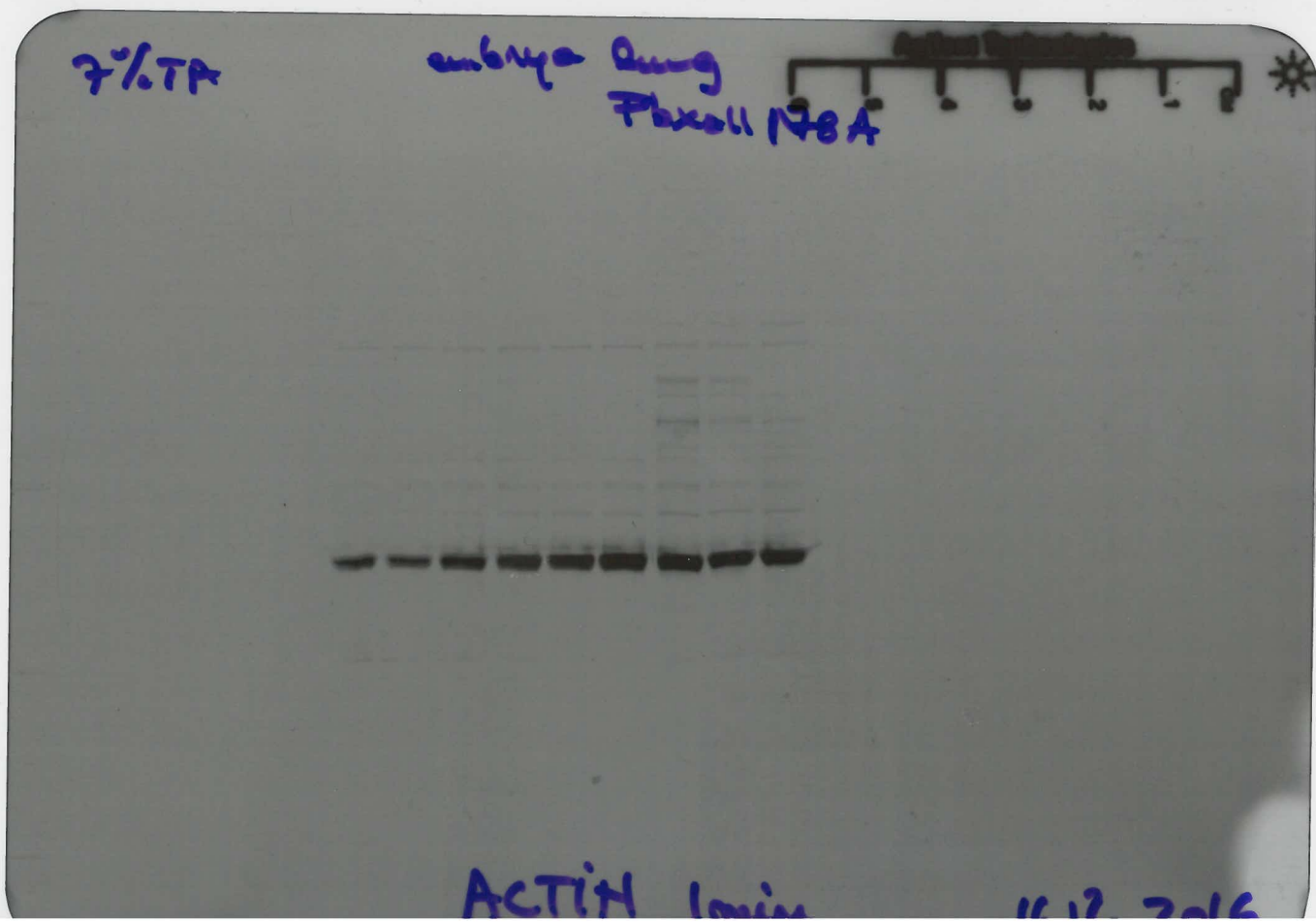

Supplement: FIGURE S1 — Original image files for the western blots included in Figure 1. [file Data_Sheet_1.PDF]
